# A framework for the design, implementation, and evaluation of output-based surveillance systems against zoonotic threats

**DOI:** 10.3389/fpubh.2023.1129776

**Published:** 2023-04-20

**Authors:** Samantha Rivers, Maciej Kochanowski, Agnieszka Stolarek, Anna Ziętek-Barszcz, Verity Horigan, Alexander J. Kent, Rob Dewar

**Affiliations:** ^1^Animal and Plant Health Agency, Addlestone, United Kingdom; ^2^Department of Swine Diseases, National Veterinary Research Institute, Puławy, Poland; ^3^National Wildlife Management Centre, Animal and Plant Health Agency, York, United Kingdom

**Keywords:** output-based, surveillance, framework, zoonotic, design, implementation, evaluation

## Abstract

Output-based standards set a prescribed target to be achieved by a surveillance system, but they leave the selection of surveillance parameters, such as test type and population to be sampled, to the responsible party in the surveillance area. This allows proportionate legislative surveillance specifications to be imposed over a range of unique geographies. This flexibility makes output-based standards useful in the context of zoonotic threat surveillance, particularly where animal pathogens act as risk indicators for human health or where multiple surveillance streams cover human, animal, and food safety sectors. Yet, these systems are also heavily reliant on the appropriate choice of surveillance options to fit the disease context and the constraints of the organization implementing the surveillance system. Here we describe a framework to assist with designing, implementing, and evaluating output-based surveillance systems showing the effectiveness of a diverse range of activities through a case study example. Despite not all activities being relevant to practitioners in every context, this framework aims to provide a useful toolbox to encourage holistic and stakeholder-focused approaches to the establishment and maintenance of productive output-based surveillance systems.

## 1. Introduction

The concept of One Health (OH) promotes the decompartmentalization of human, animal, and environmental health for more efficient and sustainable governance of complex health issues (1). This article details a framework developed as part of the MATRIX project, part of the OH European Joint Programme (OHEJP). The OHEJP is a partnership of 44 food, veterinary and medical laboratories and institutes across Europe and the Med-Vet-Net Association. MATRIX aims to build on existing resources within OH Surveillance by creating synergies along the whole surveillance pathway including the animal health, human health, and food safety sectors. This work aims to describe the design, implementation, and evaluation of surveillance systems against zoonotic threats using output-based standards (OBS).

An OBS does not strictly define the surveillance activity that must take place in a geographical area, e.g., to randomly collect and test X samples per year from Y location. Instead OBS is defined by what the surveillance system must achieve, e.g., to detect a set prevalence of a hazard with a set confidence level (2). Output-based standards therefore allow for variation in how surveillance is conducted, influenced by a variety of country/region specific factors including hazard prevalence, performance of the tests used and mechanisms of infection. These standards can also enable the comparison of results from different surveillance programs across different geographical contexts (3). Due to this flexibility, and ability to compare surveillance results across countries and sectors, OBS are useful in the OH context where animal pathogens may act as risk indicators for human health. In directing efforts to minimize spread of zoonoses in the animal population with robust surveillance, OBS may help to curtail the spread of disease at the public health level. Surveillance systems implemented using OBS will hereafter be referred to as OBS systems.

The flexibility of OBS systems also necessitates a far more involved decision-making process when designing and evaluating them. While passive surveillance can form part of the implementation of OBS, active surveillance would also be needed to ensure that surveillance is sufficient to detect the design prevalence set out in the OBS. If conducting active surveillance for a pathogen, practitioners implementing OBS have the flexibility but also the responsibility to select the most appropriate host or medium to sample from, the test type to use, and the geographical sampling distribution. They must then calculate the appropriate sample number to meet their OBS, and make sure that each of these decisions works within the practical and budgetary constraints of the existing organizational systems in their surveillance area. Guidance has already been produced for analyzing conventional surveillance systems in tools such as SERVAL (4), RISKSUR (5), EpiTools (6), and OH-EpiCap (7). And while research such as the SOUND control project is developing tools to encourage and aid OBS implementation in Europe (8), there is currently no broadly applicable, practical framework showing how OBS surveillance systems can be designed, implemented, and evaluated. In this paper we provide a framework that aims to describe the surveillance format, provide evidence-based decision-making on the best ways of applying it, and showcase methodologies to evaluate these systems using worked examples.

**Figure 1:**
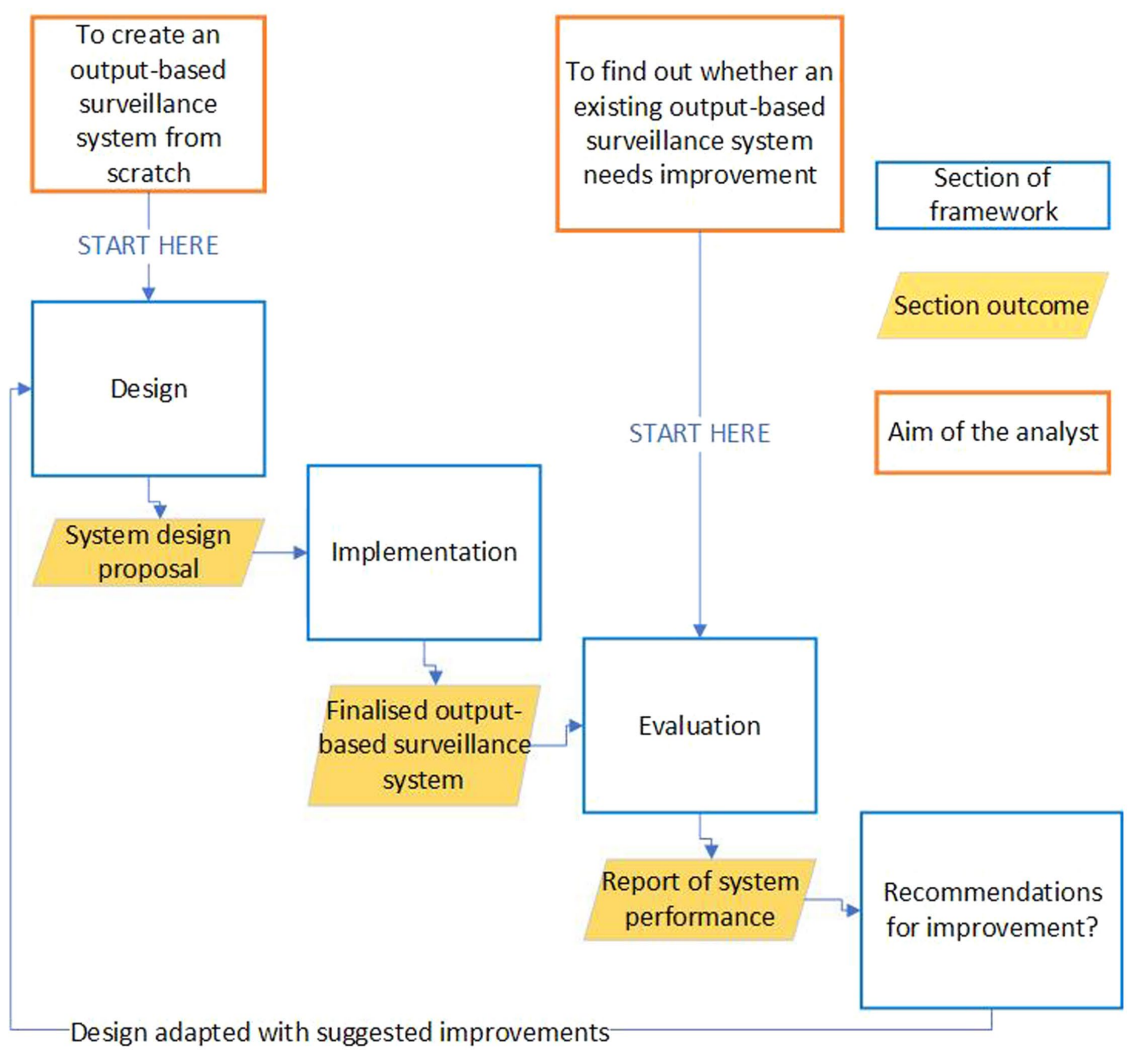
Showing the recommended route an analyst should take through this guidance if they either know they want to improve an existing surveillance system, want to design and implement an output-based surveillance system from scratch, or want to assess the performance of an existing OBS system.

This framework is aimed at those who are considering OBS as a solution to a surveillance need, whether they are looking to design and implement a system from scratch, replace a conventional surveillance system, or consider potential improvements to an existing OBS system. Not all sections may be relevant to all users. Thus, while a loose sequence exists throughout the framework, most sections can be read out of order or in isolation. Depending upon your starting point, the recommended route through this framework will differ; a diagram showing these routes can be found in Figure 1.

Throughout this framework, we will use the surveillance system for *Echinococcus multilocularis* in Great Britain (GB) as a worked example. We have chosen this pathogen because GB employs OBS for *Echinococcus multilocularis*, it is a zoonotic pathogen with a wide range of stakeholders that illustrate this process well.

## 2. Methods

### 2.1. Setting the scope of the framework

The goal of this work under the Matrix project was to develop guidelines for the design, implementation and evaluation of official controls, in this case active surveillance systems, which use OBS. This needed to include methods for:

1. Identifying operational partners and stakeholders
2. Selecting appropriate output-based systems
3. Evaluating output-based methods.

Tools for evaluating surveillance systems have already been produced such as SERVAL (4), RISKSUR (5), EpiTools (6), and OH-EpiCap (7). However, these tools do not cover all essential aspects of OBS. Hence, we wanted to produce a framework that would draw from this past work, but would focus on the practical elements of designing, implementing, and evaluating OBS systems.

### 2.2. Overarching approach

We sought to establish the essential attributes of OBS systems. For the design section, we developed a series of activities that would support the selection of appropriate design options for each attribute. The implementation section provides activities and general practical advice to assist with the roll-out of the final OBS system design. The evaluation section of the framework includes methods to assess the efficacy of the implemented design against the current context. Applying these methods would provide recommendations for improving existing OBS systems.

### 2.3. Identification of design attributes

To identify the essential design attributes of OBS systems, we drew from a literature search conducted by Horigan (9) which included a search of Scopus^1^ and PubMed^2^ using the search string “output or risk and based and surveillance or freedom” in the “title, keyword, or abstract.” This provided articles on a range of OBS systems for zoonotic and non-zoonotic hazards.

From these articles, several surveillance attributes were found to be especially important for the success of OBS systems:

1. A strong understanding of the life cycle of the target hazard. Hazard life cycles influence the selection of host species and/or medium tested for the hazard (10–13).
2. An appropriate sample number and distribution. For example, selection of risk-based, random or convenience sampling to provide a statistically robust demonstration of the hazard prevalence (10, 14, 15).
3. A sufficiently cost-effective testing approach. This influences the practical feasibility and sustainability of the system (13, 16, 17).

We then investigated the OBS system for *E. multilocularis* in GB to validate these attributes and gain further insight into these systems. Contact with the Animal and Plant Health Agency (APHA) Parasitology discipline lead and laboratory coordinator for *E. multilocularis* surveillance in GB raised three further aspects to consider:

4. The clear definition of OBS system objectives
5. The identification and engagement of key stakeholders within the system
6. The appropriate communication and reporting of results.

### 2.4. Development of framework activities

In the design section we developed activities to help ensure system designs considered these six identified attributes. These activities were mainly documentation exercises, providing an outline of the information that should be gathered and the design choices that should be made.

In the implementation section we followed systems mapping work conducted in the COHESIVE project, a partner project to MATRIX in the OHEJP. Their approach effectively described the Q fever reporting and testing system in GB (18). Recognizing the practical challenges of implementing OBS systems, we also explored project management techniques applicable to the implementation of large, complex systems, including project left-shift, integrated stakeholder feedback, and operational risk analysis and risk management, drawing practical advice from the field of systems engineering (19).

The evaluation section included activities that would provide recommendations to improve the performance of the OBS system. These were also grounded in the six OBS system attributes listed above and based on a range of previously published work and practical experience. We developed a stakeholder analysis based on work by Mendelow (20), selected because of its inclusion in the COHESIVE project (21). A methodology for cost-effectiveness analysis was also developed based on COHESIVE project outputs (22), using information gathered under a literature review of economic analysis approaches. A bespoke method for a flexibility analysis to assess how easily recommended changes to the system could be implemented was developed based on published research in the systems thinking field (23). Methodologies were also set out for evaluating the minimum required sample sizes and true prevalence of hazards in host populations using EpiTools (6), based on practical experience from the Polish *E. multilocularis* surveillance system.

## 3. Framework

### 3.1. Design of an OBS system

Primarily, design is about selecting the appropriate attributes of a surveillance system to deliver on its defined objectives, this requires information gathering, decision-making, and objective setting. Here we set out methodologies to define the:

- System objectives
- Key stakeholders
- Target hazard and surveillance stream(s)
- Sampling methods
- Testing methods and costs
- Data reporting

#### 3.1.1. System objectives

The objectives describe what the surveillance system aims to achieve from a top-level perspective, for example, to fill a regulatory requirement, to contribute to a national strategy, or to assist with disease or hazard control at the local level. Thus, the objective of an OBS system could be to demonstrate freedom from disease, or to show disease or hazard prevalence in a population with a certain level of confidence. For an OBS system the important attributes which should be considered when setting the objectives are:

- Design Prevalence: This is a fixed prevalence used to determine the hypothesis that disease/hazard is present in a population of interest (24). It can be thought of as the minimum prevalence that you would expect to detect using a given surveillance system.
- Confidence levels: This is the level of certainty that the result is correct. That is, when compared to the true level in the population, the result of surveillance would be ‘correct’ X% of the time, where X is the confidence level. The range of values for which that remains true (sample prevalence = population prevalence in X% of cases), is known as the confidence interval (25).
- Surveillance streams: these refer to the supply chain of samples from a particular host population or medium (with associated risk level) to the laboratory in which they are tested. A single hazard could have several surveillance streams. For example, the hazard could be tested for in both live animals and bulk milk from those animals, making up two surveillance streams within the one system.
- Probability of introduction: Likelihood of the disease or hazard in question being introduced to at least the number of units (e.g., animals) that would be infected given the design prevalence.

One method of compiling a list of objectives is to use a hierarchy of objectives which divides objectives into three tiers: policy, strategic, and project (26). The policy objective is the overarching reason for implementing this system at the top level such as providing confidence in disease freedom. Below this, the strategic objectives outline what needs to be achieved to attain the policy objective such as testing a specific design prevalence. Below strategic objectives are project objectives. These are the practical constraints and drivers that need to be worked within to achieve the strategic and policy objectives. Objectives in a tier below can be thought of as the ‘how’ of objectives in the tier above, while objectives in the tier above can be thought of as the ‘why’ of objectives in the tier below.

The objectives can be defined and validated through communication with the prospective system stakeholders.

Example: Great Britain must demonstrate freedom from *Echinococcus multilocularis* by upholding surveillance in accordance with an output-based scheme prescribed by the European Commission (27). Although GB has left the European Union (EU), this surveillance is still mandated by retained legislation. In this example, the policy objective therefore is to provide evidence of freedom from *Echinococcus multilocularis*. The strategic objectives describe how this OBS system aims to achieve this policy objective by detecting a 1% prevalence in a representative host population with 95% confidence, but also to do so cost-effectively. The project objectives include the sampling from appropriate definitive host(s) across a representative geographic spread, the testing using a test of appropriate sensitivity and specificity, and to do all of these within the budgetary constraints of the project.

#### 3.1.2. Key stakeholders

Stakeholders, defined as “any parties who are affected by or who can affect the surveillance system” (28), have oversight of the surveillance system and are a useful resource for informing design choices to optimize the surveillance system design.

Generally, stakeholders comprise of three distinct groups: first, governance stakeholders with the influence to set the required output of the surveillance system, e.g., a regulatory authority like the European Food Safety Authority (EFSA); second, delivery stakeholders who are actively involved in the delivery of the required outputs, such as the collection of samples, laboratory analysis or planning and strategy roles; and finally, beneficiaries who directly or indirectly benefit from the system running well, and whose wellbeing would be directly or indirectly affected by a change to the surveillance system. The general public, for example, are beneficiaries of surveillance systems involving zoonotic pathogens.

The list of stakeholders should be created based on the available information about the hazard and the objectives of the system. Once a list of stakeholders has been established, a strategy for engagement should be devised. A simple strategy could be to reach out to stakeholders using links within your network. For example, through people in your institution who have worked with them in the past. Once contact with at least one stakeholder has been established, these may then be used to establish contact with other stakeholders in the system. Following initial engagement, stakeholders can be good sources for further information gathering. A structured interview with a pre-planned series of questions is recommended.

Example: In GB, we identified potential stakeholders for the *E. multilocularis* surveillance system using literature research (particularly previous EFSA reports) and known contacts. We then contacted one of our known stakeholders to develop a wider stakeholder list. The final list, per stakeholder group, was as follows:

Governance:

- The World Organisation for Animal Health (WOAH); who record the disease status of *E. multilocularis* following the compilation of GB results.
- The GB Department for Food, Environment, and Rural Affairs (DEFRA); who compile the results.
- Local councils, who play a role in maintaining good education on the disease/hazard and responding to cases.
- The European Free Trade Association (EFTA); who advise on the measures which should be in place to control *E. multilocularis* given a change in GB’s status.

Delivery:

- The Animal and Plant Health Agency (APHA), who maintain the surveillance system, collecting samples and running analysis.
- The national reference laboratory (NRL) for Echinococcus
- APHA wildlife management team
- APHA wildlife risk modeling team.
- Veterinary practitioners, who respond to cases in dogs and hold a stake in maintaining their good health.
- UK Health Security Agency (UKHSA), who respond to and detect human cases.
- Hunters and gamekeepers, who provide carcasses from across the country for testing.

Beneficiaries:

- The Wildlife Trust, who support the welfare and environmental influences of surveillance on fox populations and the general ecology. They have a voice in ensuring surveillance does not severely, or unnecessarily, impact the wellbeing of foxes.
- Fera science, a wildlife science advice organization who receive samples from foxes and other wildlife for rodenticide survey, and who could benefit from collection of foxes for this surveillance.
- Science Advice for Scottish Agriculture, who also receive samples from foxes for rodenticide survey.
- Pet owners, who hold a stake in making sure their pets remain healthy, and who are at risk of infection in the event of incursion.
- Media outlets, who have an interest in distributing information on the quality of surveillance and in the event of case detection.
- The general public: good surveillance ensures that any incursion of *E. multilocularis* reaches as few members of the public as possible.

#### 3.1.3. Target hazard and surveillance stream

Knowledge of the hazard both informs the choice of surveillance stream, and heavily impacts the downstream practical decisions around how the system will function. Structured interviews with stakeholders along with literature research can provide knowledge about the target hazard which can be compiled into a profile. Any relevant information can be added to this profile, but it should aim to be a complete overview covering all OH aspects. If the hazard is a zoonotic pathogen, particularly if it is foodborne, this should be flagged at this stage. As with the target hazard, the choice of surveillance stream, including the target host population and/or detection medium (e.g., red fox feces or bulk milk) is key to the system design. Sampling is usually from the population considered most at risk of infection or contamination and therefore the one in which you are most likely to detect a positive case. The choice of population, and the medium from which this population are sampled, has implications on almost all areas of the workflow, including the applicable sampling types and methods, and the geographical area(s) sampled.

Example: In the case of *E. multilocularis*, the red fox is the most relevant to sample in GB as it is a definitive host for the hazard and is also widely abundant. Additionally, sampling individual animals rather than collecting environmental samples or sampling from intermediate hosts is more compatible with the available testing methods for the hazard, which require tissue samples. This also ensures that positive detection relates to one animal, rather than leaving potential for multiple sources of contamination as environmental samples would. It ensures the species and approximate location of death is known.

#### 3.1.4. Sampling methods

The distribution of the target population and the sampling strategy are essential for informing the type of test used, and how the final design proposal will be implemented.

Samples may be taken using a risk-based framework or by taking randomly from the entire population. While convenience sampling could detect a case and thereby rule out disease freedom, it is not recommended for output-based surveillance as it would be unlikely to support representative sampling of the host population to prove disease freedom. Delivery stakeholders can provide the contextual knowledge to inform the type of sampling that is most appropriate and feasible. Additional external information sources such as population surveys could provide further information to support the chosen sampling type.

Regardless of the sampling method chosen, we recommend including all populations that are relevant to the probability of introduction of the pathogen. For farmed or kept animals, this will likely include multiple surveillance streams, for example, sampling from slaughter animals, imported and moved animals. For wild animals, relevant surveillance streams may include samples from trapped or hunted animals, roadkill, resident populations, and transient or migratory populations, particularly where they cross borders.

The sampling methods link closely to the testing method chosen because the number of samples required will vary based on the sensitivity of the test used, and because certain tests will only be compatible with certain sample media (e.g., serum, nasal swab, or feces). In order to confirm the number of samples required, and to validate confidence in the test results, we suggest using a sample size calculator such as EpiTools (29).

Example: Using *E. multilocularis* in GB as an example, the red fox population was 357,000 (30). The egg flotation test can be run on intestinal tissues of fox carcasses with an estimated test sensitivity of 0.78 (31). With these inputs, EpiTools output was a suggested sample size of 383 fox carcasses to detect the hazard at a 1% design prevalence with 95% confidence, given a random sampling distribution.

#### 3.1.5. Testing methods and costs

When choosing a testing method, we suggest engaging stakeholders and reviewing literature for an overview of the tests available. From there, the most appropriate method can be chosen, considering the budget and resources available, the sensitivity and specificity of the testing method, the population available for testing and the specific surveillance scheme chosen.

As part of test selection, understanding the costs of testing helps determine whether surveillance is achievable within the budgetary constraints of your system. This is also a useful precursor to establishing which surveillance streams give the best value for money, as described in the cost-effectiveness analysis guidance in the evaluation section.

Generally, the cost of testing can be broken down into the following:

- Consumables and reagents: This covers any routine consumables costs such as reagents, PPE, laboratory, or field consumables.
- Staff: This covers all costs relating to staff, e.g., cost of staff time for sampling, testing, training and travel.
- Equipment: This covers the cost of all equipment used in the system. This may, for example, include the cost of purchasing and maintaining laboratory equipment.
- Other operational costs: This covers all other costs not accounted for, such as sample transport and equipment maintenance.

Delivery stakeholders may be able to provide detailed cost data, depending on which part of the system they are linked to. For example, laboratory stakeholders may be able to provide the procurement costs of reagents if they are already used for other tests. If further information is needed, an average price per item can be sought through the price lists of online retailers.

Example: For the GB *E. multilocularis*, we used the standard operating procedure (SOP) of the egg flotation method to generate a list of consumables, reagents and equipment which were then assigned hypothetical values detailed in Table 1.

**Table 1:** Hypothetical data showing the cost breakdown per test of the egg flotation test, and the data sources associated with these costs.

Parameter	Value	
Test	Egg flotation	
Species sampled	Fox	
Test sensitivity	0.78	
Test specificity	1	
Parameter	Unit	Cost/Value
Consumables and reagents	Per test	€56.88
Staff time (testing)	Per test	€9.26
Operational costs (excluding testing)	Annual cost	€291,593.12
Equipment	Annual cost	€894.15
Tests required at 1% prevalence	No. of tests	383
Cost of testing at 1% prevalence	Total cost	€165,823.53

#### 3.1.6. Data reporting

The types of data to report will depend on the surveillance program. In general, a system should report the frequency of data collection, the sampling strategy and testing method used, along with sensitivity/specificity, target population, sampling period and volume, methodology for results analysis, and results of testing. Commonly, these data are provided in scientific reports to the governance stakeholders.

Example: The full data reporting for GB *E. multilocularis* can be found in the annual reports produced by EFSA prior to 2021 (32), and are explored in this example.

From the 2019/2020 sampling year, GB reported results for 464 samples taken between March 2019 and January 2020, from locations across GB (31).

The testing was conducted using the egg flotation method (31) with an overview of the methodology provided in the report (32). Random sampling was used, with the sample size calculated by the RIBESS tool (33) based on the test sensitivity, and the estimated population size for detection at 1% prevalence with a 95% confidence interval. EFSA evaluated the data provided to determine whether it fulfilled the legal requirements of the legislation and assigned a disease-free status.

### 3.2. Implementation of an OBS system

To aid system implementation, it is important to outline how the proposed OBS will function in a way that communicates its vision and purpose to the system stakeholders. The stakeholders can then provide feedback on the proposed system design and suggest improvements to make it more practically or economically viable. Once the design has been agreed, a strategy can be devised for maintaining the continued quality of the system through test validation and accreditation.

#### 3.2.1. System mapping

System mapping provides a flow diagram showing all processes from the point of sample collection to the reporting of results. Visualizing the entire system in this way helps document the sequence of the surveillance system and makes the function of the system easily disseminated.

The simplest method for system mapping is constructing a flow diagram with direct input from your stakeholders (18). This should describe the steps from sample acquisition to result analysis. Most of the system structure will already have been determined in the design process. However, any remaining aspects of the system that are unclear should be highlighted in this flow diagram and clarified by the stakeholders. The diagram should outline which stakeholders will be involved at each step in the process.

**Figure 2:**
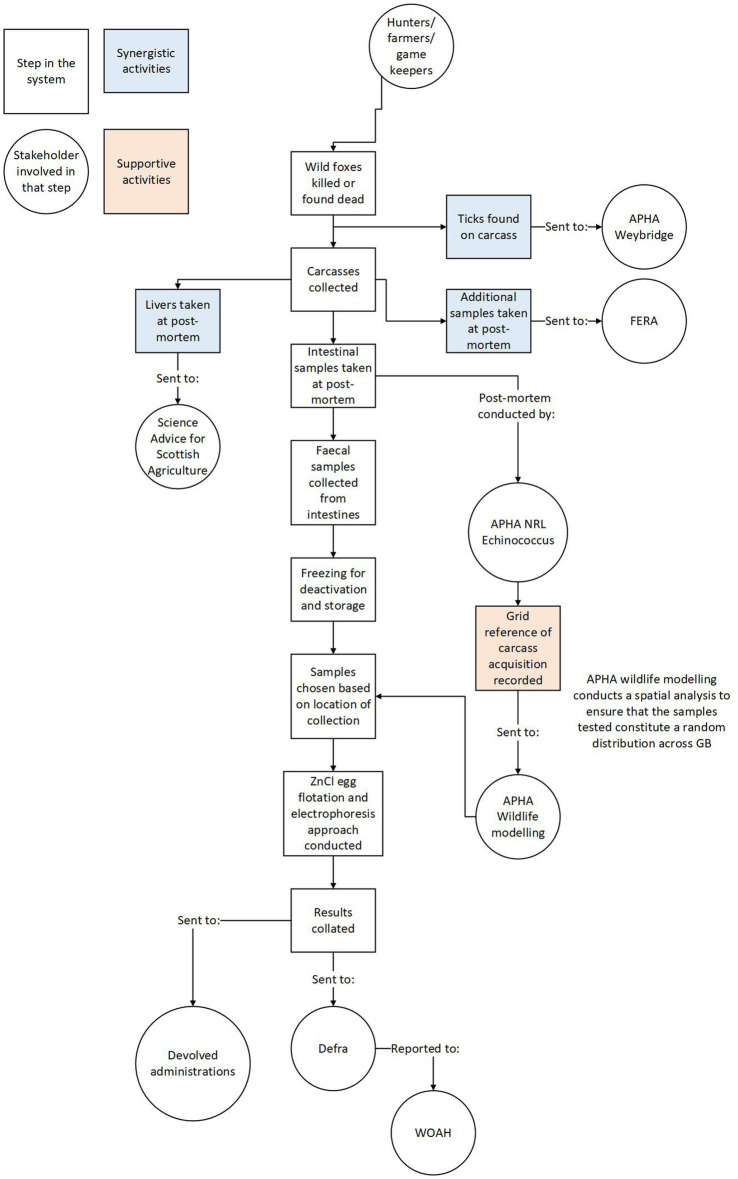
Showing the system structure and chronology from carcass collection to result reporting. Rectangles represent steps in the system while circles represent stakeholders involved in relevant steps.

The system structure map can also be used to represent any synergistic systems linked to the surveillance, for example, if the same samples could be used for other purposes. This helps document the linkages of the surveillance system with other activities and highlights opportunities to make sampling more practical, cost-effective and mutually beneficial. The surveillance system for *E. multilocularis* in GB, for example, has multiple stakeholders each contributing to, and benefitting from, its various stages (Figure 2).

#### 3.2.2. Project management planning

Effective project management is required to coordinate the implementation of your proposed surveillance design, especially if operating to a deadline. Formal training in this field is highly recommended before undertaking the implementation of any large, complex output-based surveillance systems. However, we suggest drawing ideas from systems engineering practices such as project “left shift.” This focusses on shifting project funding and input to the start of a project rather than the end of it. Early investment in a project provides better value for money due to inflation. Also, spending more time on the early planning stages of the project can prevent mistakes that may be challenging or expensive to resolve later in the project (34).

In the implementation of output-based surveillance systems, left-shift means investing heavily in building up the cohesion and experience-base of the delivery stakeholders of the system. These are similarly highlighted as important factors in the RISKSUR framework best practices (35). This could include investment in dedicated training for sample collection, analysis, and result reporting, or a pilot, where a small number of samples are collected and tested to ensure all aspects of the system work well together before scaling up. Outreach could be part of this early investment. For example, allowing laboratory staff time to shadow sample collectors and vice versa. Such activities will greatly improve cohesion along the sample analysis pipeline, allowing stakeholders to form close working relationships, facilitating a faster response to problems and potentially contributing to efficiency gains as stakeholders share experiences with one another.

Verification and validation stages with stakeholders during implementation are also recommended. These stages could test whether each part of the system delivers on the original system objectives and provides value to stakeholders as the systems are being implemented (36). Verification, as with all stages of project management, should be well documented and we recommend having a robust documentation process to make sure plans and activities are transparent to the implementation team and wider stakeholders (37–40).

Another recommendation is to conduct an operational risk analysis. This can identify, assess, and derive actions against issues which can occur during the implementation process. In this risk analysis, the probability of each of these risks occurring and the impact if these risks occur as either Low, Medium, or High. This facilitates decision-making on the proportionate action to take to either avoid these risks, mitigate their impacts, or accept them. We recommend guidance in Lavanya and Malarvizhi (41) or the textbook by the Institution of Civil Engineers (42) for further details on the steps to follow for operational risk analysis. All changes made to avoid a risk must be checked against the prior design stages and documented.

Stakeholders should agree with the outcomes of risk analysis, to any resultant changes to the system design and any accepted risks. Agreeing the final system design and implementation strategy with delivery stakeholders will improve the likelihood of successful implementation (43).

### 3.3. Evaluation of an OBS system

This section provides a range of evaluation exercises to help direct improvements to existing OBS systems.

#### 3.3.1. Evaluation of system objectives

This evaluation determines whether the system objectives are still relevant and complete. For example, the hazard prevalence may have changed since the implementation of the OBS system, so is the design prevalence for detection still appropriate? A new test may have been developed for the target hazard, so how does this compare with the test currently implemented?

Assessing the suitability of the system objectives requires analysis of current research relevant to the OBS system. This can be conducted through a combination of literature review and stakeholder engagement, to explore the following questions:

- Has the level of detection changed since the first implementation of the surveillance system? Has prevalence of the hazard increased/decreased or changed in its geographical distribution?
- Has new evidence come to light on the dynamics of the hazard under surveillance? For example, have new competent hosts been found?
- Have new tests been developed for the same hazard and host as the original surveillance system? Do these new tests offer improved sensitivity and/or specificity to the current option; do they offer other advantages?
- Have any aspects of the surveillance system been recognized to be operating particularly well? For example, have other groups taken inspiration from the current system and implemented the same methods elsewhere?
- Have any issues or doubts about aspects of the surveillance system been raised? Are any of these corroborated by data?
- Has the political or legislative context of surveillance changed? Has the target hazard or population become higher or lower priority to governing bodies? Is the need for surveillance brought in to question by these changes?

#### 3.3.2. Flexibility analysis

It is expected that every system will undergo changes throughout its lifecycle. A good output-based surveillance system needs to be adaptive to technological, practical, or political changes to continue delivering value for its stakeholders. A flexibility analysis determines how changes to a system could affect its various stakeholders and its ability to deliver on its core objectives.

Determining the flexibility of the system requires systems thinking so we recommend using causal loop diagrams to illustrate links between system components and stakeholders. The system components are any aspect of the system that affect its overall function. The surveillance streams, test type, number of tests, design prevalence, and even the method of result reporting and analysis can all be considered system components. Causal loop diagrams illustrate the dynamics of complex systems by showing the positive or negative relationships system components have on one another and on the stakeholders (23, 44). To produce these diagrams, the first step is to identify which system components affect each stakeholder. For example, sample collectors will be directly impacted if they are asked to collect more samples. The number of samples required is influenced by the sensitivity and specificity of the test chosen, and by the design prevalence and required confidence level set out in the system objectives. Hence, these stakeholders are *linked* to the sampling requirements, the test chosen, the design prevalence, and the required confidence in the results. When a link is demonstrated, it is essential to show whether the relationship is positive or negative. For example, higher test sensitivity has a negative effect on the number of tests required since more sensitive tests are statistically more likely to detect a hazard if it is present. Logically, the number of tests required positively influences the number of samples taken: more tests required means more samples will need to be taken and consequently, these too are linked. While making these links, it is likely that further interrelationships between different stakeholders and system components will emerge. Documenting all relevant links will provide a complete picture of the emergent impacts of design decisions on each of the stakeholders.

Once the links between design decisions and stakeholders have been established, engagement of stakeholders is required to determine their tolerance to change. If stakeholders operate under fixed constraints these should be identified and documented. For example, delivery stakeholders may be working within a budgetary range. If they can agree to an increase in sampling rate, what is their maximum sample number? Governance stakeholders may have some tolerance in the design prevalence or testing confidence they expect to see from a surveillance system. What is this tolerance and to what extent could the system adapt before those tolerances are exceeded?

**Figure 3:**
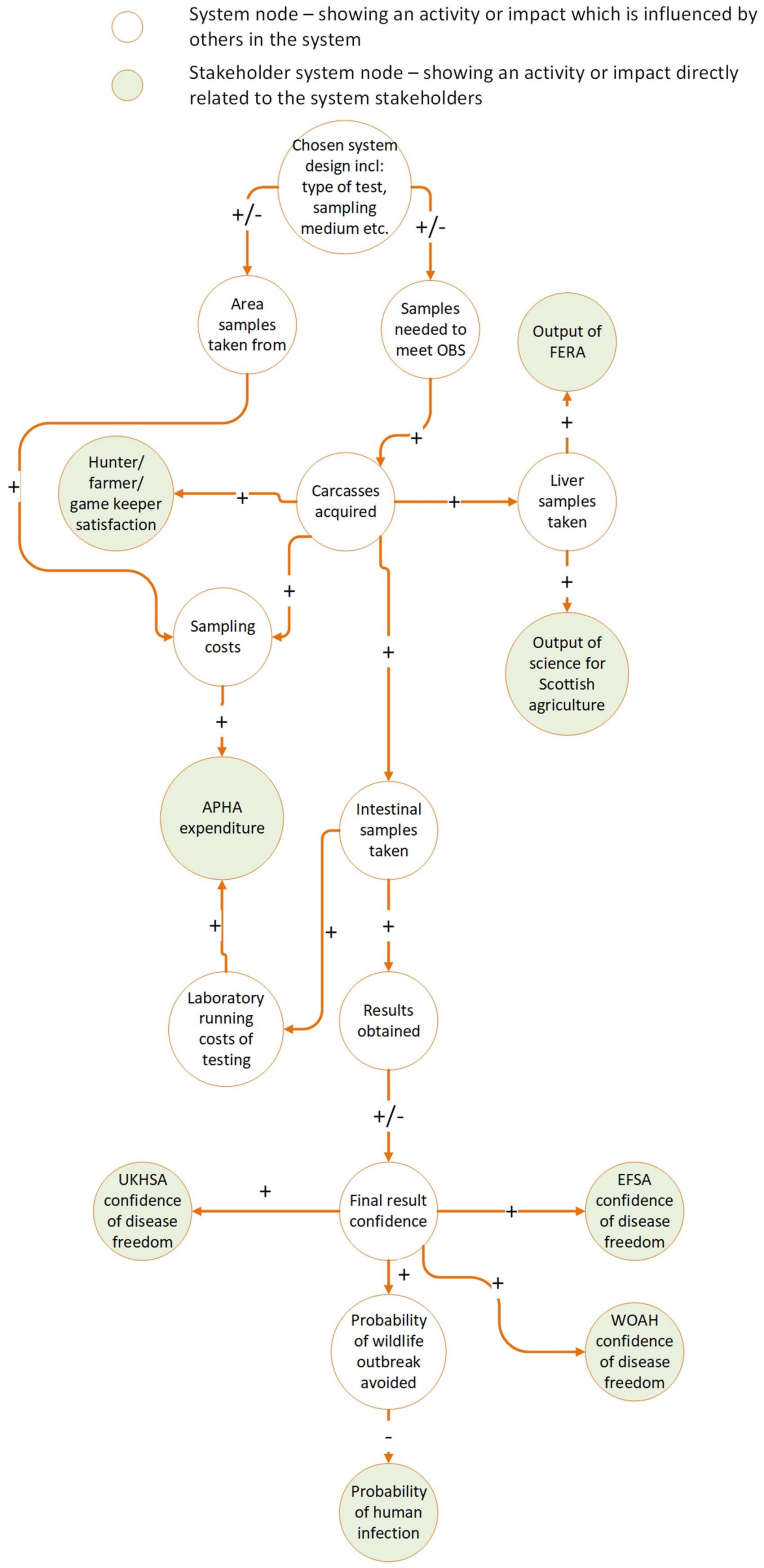
Example causal loop diagram illustrating the positive and negative interrelationships of different parts of the UK *E. multilocularis* surveillance system and the perceived stakeholder benefits and losses from changing aspects of the system.

Example: For *E. multilocularis* surveillance in GB, we determined that changing the type of surveillance scheme, for example the test used, would impact the required sample size, and thereby affect both the workload of the delivery stakeholders and the confidence in the test results, altering the outcome for end beneficiaries. By representing the system using a causal loop diagram (Figure 3), we identified 5 distinct interrelationships to be aware of if any changes to the system are considered. These were:

- The chosen surveillance scheme will affect how many carcasses are collected, and where they are collected from (for example, if collected according to risk-based sampling rather than random sampling). This has ripple effects on every other part of the system.
- A higher sample requirement would mean more time and money spent collecting those samples. It would also demand more from farmers, hunters and gamekeepers to provide carcasses for analysis. This could strengthen or damage relationships with these stakeholders, depending on their appetite for collaboration, and thereby increase or decrease their satisfaction with the system and their willingness to supply samples (45). Hunters, farmers and gamekeepers already deliver an excess of samples to APHA, and it was estimated they would be receptive to an increase in the number of carcasses asked of them if needed, though their specific upper-bound tolerance was unknown.
- More carcasses collected means more of all sample types are available for commercial collaborators.
- A higher sampling rate, or improvement in the geographical spread of collected samples will increase the overall confidence in the surveillance system. It will increase the probability that cases in wildlife will be detected before the disease becomes established in the wild population. This will reduce the number of human cases, and therefore provide a higher benefit to society at large.
- A change in the costs of maintaining the system, and the downstream effects on the benefit to stakeholders, will affect the benefit–cost ratio of the surveillance system. A higher benefit–cost ratio means the surveillance system generates greater value for money.

#### 3.3.3. Stakeholder analysis

This evaluation determines and depicts the level of interest and influence current stakeholders have in the system. Stakeholders have diverse views and roles. Thus, to understand them, it is a useful exercise to categorize them in order to identify the most influential stakeholders, or those who hold the largest stake in the system achieving its objectives. As a result, it is then possible to establish whether the position of individual stakeholders on the matrix is appropriate. A modified Mendelow matrix is an effective way to categorize stakeholders. This is a two-dimensional matrix plotting the interest and influence of stakeholders (20). It provides information about which stakeholders are the most engaged, and which are most influential.

Structured interviews should be used to determine the level of influence and interest in the system. Direct questions are a good starting point, for example ‘what is your perceived level of influence on the system?’. It can be useful to follow up with more descriptive questioning. A question which asks the stakeholders how they might implement change to a system could return more tangible insights into the barriers stakeholders face when trying to implement change. A stakeholder with high influence will likely have a strong idea of how to enact change to the system and may even have been directly involved in making prior changes to the system.

The level of interest in the system involves how stakeholders would be affected by changes to the system. When ascertaining the interest of stakeholders, questions that explore hypothetical scenarios may yield richer results, for example, asking how a stakeholder might be affected by increasing or decreasing the sample numbers taken, or by changing the objectives of the system. If their answers indicate they would need to take immediate action because of these changes, this illustrates a high level of interest in the system. For beneficiaries of output-based surveillance systems, such as the general public, who may not be aware of the implications of changes to it on their own health and wellbeing however, this can be a challenge. A judgment can be made in these cases based on the prior information compiled.

Another tool for collecting information from stakeholders could be survey-based questions rating interest and influence on a quantitative scale, for example from 1 to 10. With interviews and surveys, every effort should be made to contact as many stakeholders as possible from across the system. Where this is not possible, a proxy can be used to evaluate/assess the influence and interest these stakeholders have. This could be based on the perceptions of other stakeholders in the system, taking care to get input about missing stakeholders from as many other stakeholders as possible. Once the bulk of information has been compiled, they can be placed on the Mendelow’s matrix. A completed matrix of all stakeholders should then be verified by the stakeholders.

Finally, you should evaluate whether the position of the stakeholders on the matrix is still appropriate, particularly regarding the influence they have on the system. This can be assessed by asking stakeholders whether they think they should have more or less influence on the system in the future. A desire to change their level of influence can be represented on the matrix with arrows. Arrows provide an indication of stakeholder satisfaction and suggest areas for improving stakeholder involvement.

Example: For the *E. multilocularis* surveillance system in GB, we reached out to stakeholders *via* email or through interviews, assembling information to plot these stakeholders on a Mendelow matrix. We interviewed the following stakeholders:

- APHA Parasitology discipline lead and laboratory coordinator for *E. multilocularis* surveillance in Great Britain.
- Carcass collection coordinator for *E. multilocularis* surveillance in GB.
- APHA discipline lead for wildlife epidemiology and modeling, leading *E. multilocularis* sample selection, and risk modeling.
- Science Advice for Scottish Agriculture research coordinator, rodenticide sampling in wildlife
- Fera Science research coordinator, rodenticide sampling in wildlife

Additionally, we contacted the UK Health Security Agency Emerging Infectious Zoonoses Team and DEFRA *via* email but were unable to reach WOAH. When interviewing, we discussed the following topics with each stakeholder:

- The role of the stakeholder within the system
- The perceived roles of other stakeholders in the system
- Their perceived understanding of how the surveillance system practically functioned to deliver outputs
- Their perceived influence on the system
- Their satisfaction with the system, particularly with regards to the level of influence they had on it.

**Figure 4:**
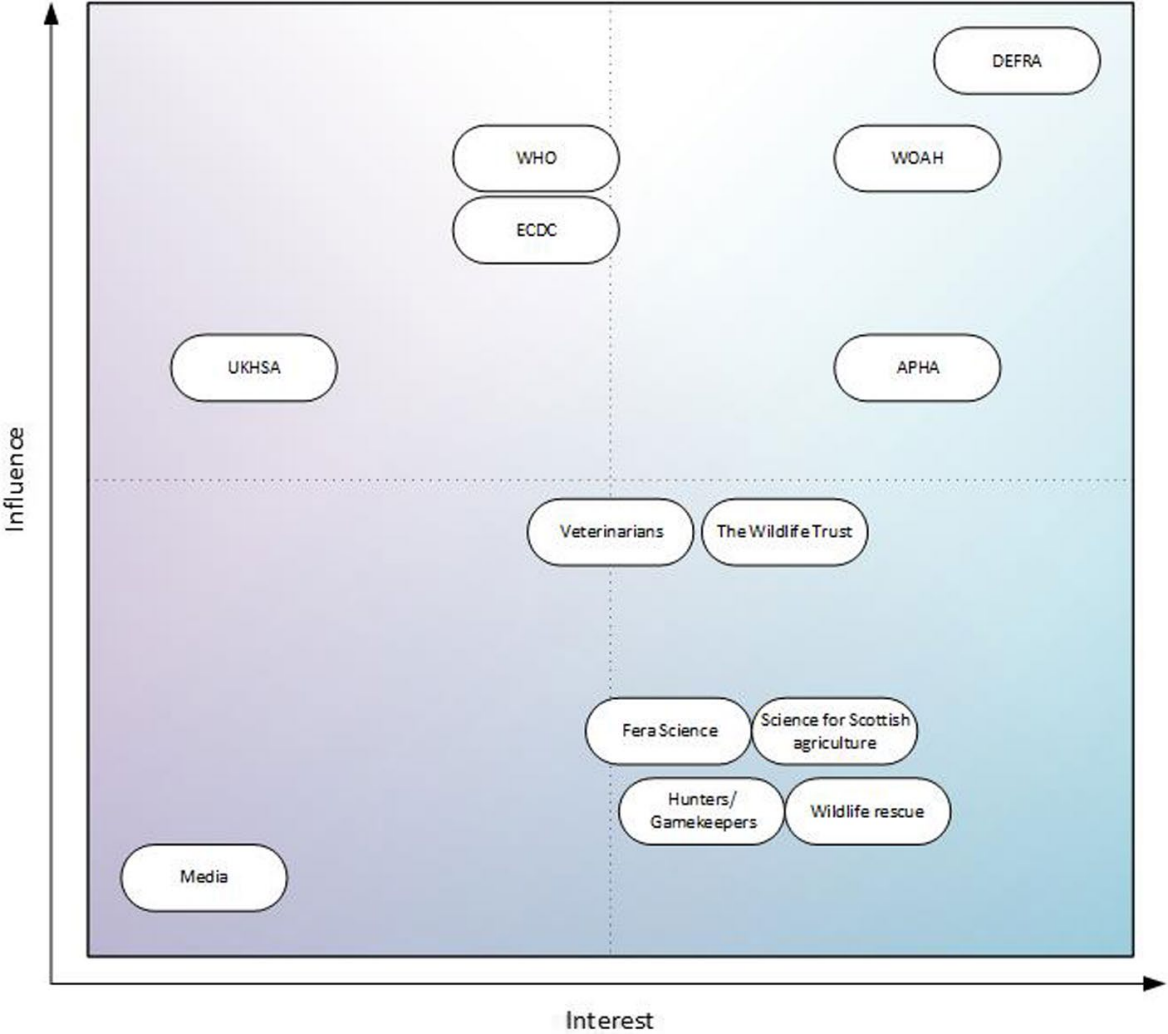
Stakeholders involved in GB *E. multilocularis* surveillance mapped to a Mendelow matrix, sorted by level of influence and interest in the surveillance system.

For stakeholders that could not be contacted directly, attributes were estimated from the expert knowledge of the other stakeholders; from their past interactions with these stakeholders and their experience working within the system. With the information compiled in the interviews, it was possible to map each stakeholder on a Mendelow matrix (Figure 4).

In the future, DEFRA will receive the annual reports of the surveillance, therefore, they have both high interest and high influence on the matrix. APHA, and WOAH are also in this quarter of the matrix; APHA are responsible for carrying out the surveillance and WOAH are responsible for producing the annualized reports to prove disease freedom and publishing results shared by member states. With the current GB situation for *E. multilocularis*, the UKHSA is in the low interest, high influence quarter of the Matrix. However, this would likely change to high interest, high influence, if there were changes to the status of *E. multilocularis* in GB. When asked, satisfaction was very high: no stakeholder felt they needed more or less influence on the system.

#### 3.3.4. Minimum sample size evaluation

This evaluation calculates the minimum sample size required to detect hazard at a set design prevalence and confidence level. This calculation is relevant for monitoring the hazard in the population. If the sample size is too big it will result in excess financial cost. If the sample size is too small, it can lead to the system not achieving its objectives. Scientific publications, international and governmental statistical data, hunting associations or other professional organizational data, expert opinions, and gray literature can all provide relevant population size data and information about test sensitivity. Furthermore, the sensitivity of the test can also be determined *via* validation studies and in the case of a commercial test, *via* the test manufacturer. This information can then be used to calculate the minimum sample size needed for surveillance using the online EpiTools calculator - “Sample size for demonstration of freedom (detection of disease) in a finite population” (29).

This tool can calculate the sample size needed to achieve the required probability of detecting disease or presence of a hazard (herd-sensitivity) at the defined design prevalence for a finite population, assuming a diagnostic assay with known sensitivity and 100% specificity. These calculations use an approximation of the hypergeometric distribution (29, 46). According to MacDiarmid (46) the probability (β) that there are no test-positive animals in the sample tested can be calculated as:


$$\beta ={\left(1-\frac{n\;SE}{N}\right)}^{pN}$$

where:

- *p* = true prevalence of infection
- SE = sensitivity of the test
- *N* = herd size
- *n* = sample size

The required parameters (inputs) for the calculator are:

- Population size
- Test sensitivity
- Desired herd-sensitivity
- Design (target) prevalence

The main output of this EpiTools analysis is the number of samples required to provide the desired herd sensitivity for a specified design prevalence. The results of this analysis are 383 sample required for both the SCT and IST, and 336 samples required for the PCR. The calculations concerned *E. multilocularis* in the red fox population in selected European countries. In these calculations, the EpiTools calculator inputs were set as follow:

- Red fox population size - defined according to the data from publications and reports (Table 2)
- Sensitivity of *E. multilocularis* detection test (sedimentation and counting technique (SCT) 0.78, intestinal scraping technique (IST) 0.78, or PCR method)- derived from publications and reports as reported in Table 3.
- Desired herd-sensitivity – was set at 0.95
- Design (target) prevalence – here was set in accordance with the calculated true prevalence

**Table 2:** Calculation of the number of samples required to detect *E. multilocularis* in the red fox population in selected European countries.

Country	Red fox population								Sample size for demonstration detection of disease						
	References	2009	2010	2011	2012	2013	2014	2022	2009	2010	2011	2012	2013	2014	2022
Poland	[1]				193,402	210,332	198,679					19	19	19	
Latvia	[2]	35,000	34,800						9	9					
Denmark	[3]						31,100							405	
Hungary	[4]							78,000							60
Romania	[5, 6]		53,292							63					
Finland	[7, 8]						150,000							384	
Ireland	[7, 9, 10]						150,000							339	
Great Britain	[7, 11]						240,000							353	
Norway	[7, 12]		70,000		70,000	70,000	151,000			475				476	

References: [1] – The Forest Data Bank (47); [2] – Kirjušina et al. (48); [3] – Danish Centre For Environment And Energy (49); [4] – European Health and Digital Executive Agency European Food Safety Authority (50) (HaDEA); [5] – Şuteu et al. (51); [6] – Romanian National Institute of Statistics (52); [7] – European Food Safety Authority (50); [8] – Kauhala (53); [9] – Hayden and Harrington (54); [10] – Marnell et al. (55); [11] – DEFRA Department for Environment and Agency (56); [12] – Sviland et al. (57).

**Table 3:** Calculation of the true prevalence of *E. multilocularis* in red foxes in selected European countries.

Country	Apparent prevalence calculation					True prevalence calculation				
	Survey references	No. of tested samples	Number of positive results	Method	Apparent prevalence (%)	Sensitivity and specificity references	Method sensitivity	Method specificity	True prevalence (%)	95% CI
Poland	[1]	1,546	255	SCT	16.5	[12]	0.885	1	18.64	16.64–20.82
Latvia	[2]	45	16	SCT	35.6	[12]	0.885	1	40.18	26.24–56.68
France	[3]	3,307	562	SCT	17	[12]	0.885	1	19.2	17.8–20.69
Germany (northern)	[4]	3,094	523	SCT	16.9	[12]	0.885	1	19.1	17.65–20.64
Denmark	[5]	546	4	SCT	0.73	[12]	0.885	1	0.83	0.32–2.11
Hungary	[6]	100	5	SCT	5	[12]	0.885	1	5.65	2.43–12.63
Romania	[7]	561	27	IST/SCT	4.8	[13]	0.78	1	6.17	4.27–8.86
Belgium	[8]	990	243	IST	24.55	[13]	0.78	1	31.47	28.16–35.03
Slovakia	[9]	660	49	IST/SCT	7.4	[13]	0.78	1	9.52	7.26–12.41
Estonia	[10]	17	5	SCT	29.4	[12]	0.885	1	33.23	15.01–60.04
Finland	[11]	265	0	PCR	0	[11]	0.78	1	0	0–1.83
Ireland	[11]	331	0	SCT	0	[12]	0.885	1	0	0–1.3
Great Britain	[11]	434	0	PCR	0	[11]	0.85	1	0	0–1.03
Norway	[11]	523	0	PCR	0	[11]	0.63	1	0	0–1.16

References: [1] – Karamon et al. (58); [2] – Bagrade et al. (59); [3] – Combes et al. (60); [4] – Berke et al. (61); [5] – Enemark et al. (62); [6] – Sréter et al. (63); [7] – Sikó et al. (64); [8] – Hanosset et al. (65); [9] – Bagrade et al. (59), 2001; [10] – Moks et al. (66); [11] – European Food Safety Authority (50); [12]–(67); [13] – Hofer et al. (68).

**Figure 5:**
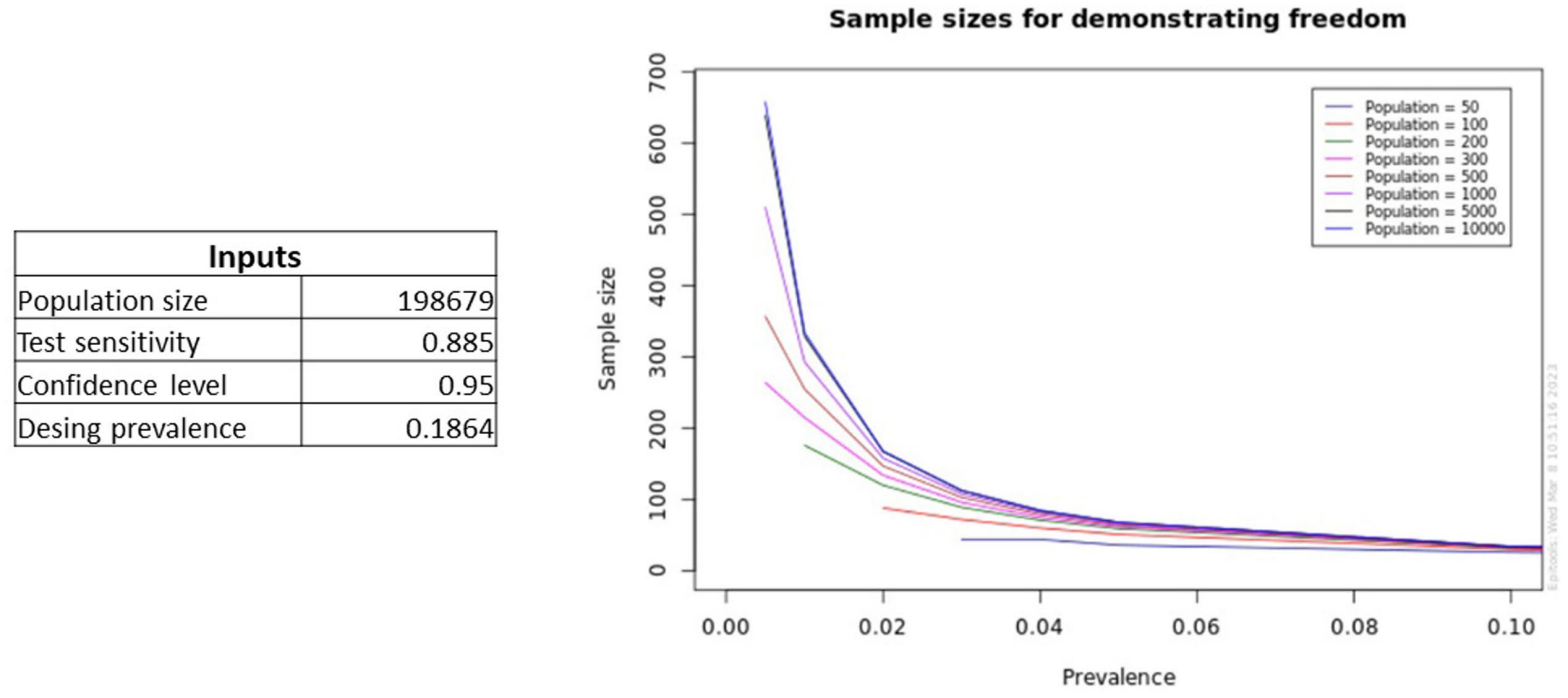
Plots generated by the EpiTools calculator showing predictions for different prevalence levels and population sizes for a specified test sensitivity.

Furthermore, this EpiTools calculator can generate graphs of the sample sizes needed to achieve the desired herd sensitivity, for a defined test sensitivity and range of population size and design prevalence (Figure 5).

#### 3.3.5. True prevalence evaluation

This section estimates the true prevalence to confirm or correct any previously calculated prevalence of disease (apparent prevalence). Most diagnostic tests have imperfect sensitivity and specificity. Calculation of true prevalence (the proportion of a population that is actually infected) considers the sensitivity and specificity of the applied test. Calculating the true prevalence can determine whether the choice of design prevalence for the system is still appropriate. This is more accurate than calculations of apparent prevalence (the proportion of the population that tests positive for the disease) which are reported in the majority of epidemiological studies/reports and do not include these parameters. Scientific publications, international and governmental reports, expert opinions, and gray literature can all be used to find these data.

A useful tool for calculating true prevalence is the EpiTools calculator – “Estimated true prevalence and predictive values from survey testing” (29). This tool calculates the true prevalence, as well as positive and negative predictive values, and likelihood ratios based on testing results using an assay of known sensitivity and specificity (29). For example, true prevalence of *E. multilocularis* in Poland was calculated by EpiTools calculator as 18.64% (95% CI, 16.64–20.82) while apparent prevalence was 16.5%. Based on this example one can see that number of tested samples, number of positive results, method sensitivity as well as method specificity effect on calculation result. For Poland and other selected countries of EU calculations of true and apparent prevalence are presented in Table 3. Furthermore, EpiTools calculator enables graphical visualization of output results.

Using *E. multilocularis* prevalence in Poland as an example, the inputs required to perform computations by the EpiTools calculator are as follows:

- Number of examined samples obtained from red foxes (intestines or faeces samples) and number positive samples - set according to data from publications and reports as indicated in Table 3.
- Sensitivity and specificity of the method (SCT, IST or PCR method)
- Confidence level – was set at 0.95
- Type of confidence interval for apparent prevalence – Wilson CI was used
- Type of confidence interval for true prevalence – Blaker was used

To determine the true prevalence (TP) from these data, EpiTools applies the Rogan-Gladen estimator, using the following formula:


$$TP=\frac{\left[AP+\left(SP-1\right)\right]}{\left[SP+\left(SE-1\right)\right]}$$

where:

- AP = apparent prevalence
- SP = specificity
- SE = sensitivity

#### 3.3.6. Cost-effectiveness analysis

It is important that the testing process and the overall cost of the wider surveillance scheme is as cost effective as possible. This likely also affects stakeholder satisfaction and may affect the long-term sustainability of the system. To evaluate this, it is recommended to carry out a cost-effectiveness or cost–benefit analysis (or similar applicable economic analysis method). This example specifically looks at cost effectiveness analyses (CEA).

Cost effectiveness analyses measure the input cost required for the system to produce a given output. Unlike some other economic analysis approaches, the ‘effectiveness’ component of a CEA can be defined by the analyst. In output-based surveillance, the output is already defined at the operational level (to detect a stated design prevalence with a stated confidence). Cost effectiveness analysis can easily be applied in these cases, to measure the cost input required to meet these outputs. This can then be compared directly to alternative approaches. Gathering data on the cost inputs of a system first requires an inventory of all materials and reagents used, staff time required, and any transport and sample collection costs. Materials and reagents can be found using laboratory standard operating procedures (SOPs). The price of each cost component may be attainable through contact with stakeholders working within the system. Alternatively, these may be found on supplier websites. Staff time should ideally be derived through contact with the staff themselves, preferably staff who have a holistic view of the system from sample acquisition to result reporting.

When collecting data on alternative test types which are not yet in use, it may be useful to use proxies. Proxies can be similar tests already conducted for other pathogens, and hence already have internal costs listed in the organization. Data on alternative tests may also be found on supplier websites. Every test type will be different so it’s important to avoid biases wherever possible. For example, if you are calculating costs over a year and a piece of key equipment needs maintenance every 4 years, then this cost needs to be considered fairly: it should not be ignored but should also not be considered in full for a single year of testing. A fair solution would be to divide this cost by the years between maintenance activities to make it a normalized annual cost output.

Data for each testing type must be calculated per test and multiplied by the required sample size based on the sensitivity of each test. This can be calculated using the EpiTools online resource. Doing so allows for direct comparison between the cost-effectiveness of each test type.

Example: In the design section, in test costing, we used hypothetical data as an example of the cost of the egg flotation test for *E. multilocularis* surveillance. An objective for this surveillance is to ensure that the system uses a method that is practically and financially feasible. This can be conducted by comparing the costs of the current testing method against the known surveillance budget. However, only a comparison of multiple surveillance design options can optimize value for money. For *E. multilocularis* we produced a CEA comparing the hypothetical costs of multiple testing methods; the egg flotation test, and two alternate methods identified in the sampling methods section. When working with estimated costs, the CEA can be used iteratively to generate a range of outputs or, if the upper and lower bounds of cost data are known, then this can provide a minimum and maximum cost for the surveillance.

Cataloging the other tests available was conducted through discussions with the stakeholders and through literature research. The annual EFSA report on *E. multilocularis* surveillance in Europe was an essential resource, summarizing how each country in Europe was conducting their tests, describing a range of alternative test-types (69).

We identified two alternative methods, the SCT and a real-time PCR method. APHA conducts the SCT as part of the external quality assurance and proficiency testing schemes provided by the European Union Reference Laboratory for Parasites (EURLP) for the detection of *Echinococcus* spp. worms in intestinal mucosa. The instructions and procedure provided by the EURLP for this testing was used to broadly determine the consumables, reagents and equipment required for this test (70). Prices per test were generated using hypothetical data. The staff time spent processing samples, ‘lab time,’ was calculated using an average sample throughput of 15 samples per day based on information from literature (71). The additional time costs including sample collection and post-mortems (‘non-lab time’) were assumed to be the same for all methods, and therefore are set at a blanket cost per sample (hypothetical data).

The real time PCR method used in this evaluation is the QIAamp Fast DNA Stool Mini Kit (QT) combined with a TaqMan PCR, the method for which has been previously described in literature (72, 73). A combination of this literature, and in-house SOPs were used to populate a list of consumables, reagents, and equipment (74) which were then assigned hypothetical costs.

The SOPs and information gathered for these tests were used to create the consumables, reagents and equipment lists. Each component was then assigned a hypothetical cost. Costs for two alternative methods of testing previously identified were also produced based on protocols found through literature searches, and the three methods were compared in a cost effectiveness analysis (Table 4). Hypothetical values were also generated for staff time, sample transport and post-mortems. All cost values were then added together to provide the annual costs of maintaining a surveillance system using each test type, including the costs for sample collection, post-mortem, testing, and epidemiological services linked to the system.

**Table 4:** Showing the cost-effectiveness of three different testing methodologies for *E. multilocularis* at detecting a 1% prevalence detection with 95% confidence (hypothetical data).

Parameter	Unit	Test		
		Egg flotation	SCT	qPCR
Species sampled	–	Fox	Fox	Fox
Throughput	–	Batch of 20 every 12 h	10–20 per day (Average 15)	12–30 min per sample (Average 21)
Test sensitivity	–	0.78	0.78	0.89
Test specificity	–	1	1	1
Consumables and reagents	Per test	€56.88	€3.74	€12.48
Staff time (testing)	Per test	€9.26	€17.57	€10.32
Operational costs (excluding testing)	Annual cost (800 tests)	€291,593.12	€291,593.12	€291,593.12
Equipment	Annual cost	€894.15	€625.05	€18,860.40
Tests required at 1% prevalence	No. of tests	383	383	336
Cost of testing at 1% prevalence	€	€165,823.53	€150,408.31	€148,989.54

The total annual cost for each testing methodology was converted into a mean cost per test. The number of samples to be taken was calculated using EpiTools, an online sample size calculator developed by AUSVET (6) with the test sensitivity, design prevalence, confidence level and host population size as inputs. Since positive results were assumed to be followed up and confirmed, the specificity of all tests was set to 1. The test sensitivity of 0.78 for the zinc egg flotation (EF) and SCT methods is the value recommended for use by EFSA for this type of testing, whereas test sensitivity for the qPCR method is the average of those sourced from literature. From these data the qPCR is the most sensitive of the testing methods with a sensitivity of 0.89.

The minimum number of tests required to detect a 1% prevalence with 95% confidence with the sensitivities specified by these tests was then multiplied by the cost per test to provide the overall cost of each testing methodology.

The costs of each methodology were compared. For annualized costs, such as sample collection and post-mortem, the per test cost was calculated based on the approximate number of samples collected in GB for the sampling year 2021–2022: 800 (75). This was multiplied by the number of tests required, determined using the EpiTools calculator.

For this hypothetical scenario, the SCT is the most economical when it comes to consumables and reagents, costing an estimated €3.74 per test compared to the €12.48 and €56.88 required for the PCR and EF, respectively. This is also true for the estimated annual cost of equipment and maintenance, with the SCT requiring an estimated €625.05 per year compared to €894.15 for the EF and €18.860.40 for the PCR equipment. This difference is mainly due to the comparatively large maintenance cost for real time PCR equipment. Where these outputs differ, however, is the cost of staff time associated with each test. We estimated the cost-per-test of both the EF and PCR at between €9–11 whereas due to the time intensive nature of the SCT, the per cost test was determined to be €17.57 based on staff processing an average of 15 samples per day (71).

Overall, with this model the qPCR is shown to be the most cost-effective testing method due to its lower number of tests required per year.

#### 3.3.7. Propose improvements to the system (if applicable)

Each evaluation from the previous section will have developed an understanding of how well the surveillance system currently functions. This may have highlighted areas where the surveillance system needs improvement. Improvements do not necessarily mean increases in testing output, but rather changes to the system that make it more effective at achieving its objectives at the time of evaluation.

Examples of potential improvements include changes to test type to increase cost-effectiveness or accuracy of surveillance, changes to design prevalence to detect a higher or lower population prevalence with greater confidence or changes to sample number to better reflect the chosen design prevalence.

Any proposed improvements to the system constitute a change to the design proposal of the surveillance system. Hence, it may be necessary to go through the stages of design and implementation to ensure improvements are properly considered from all angles by the relevant stakeholders.

## 4. Discussion

Output-based standards can allow for variation in surveillance activities to achieve a universal objective and may be useful in the OH context where surveillance for animal pathogens can act as risk indicators for human health. In addition to the context of zoonotic pathogens, OBS may also be useful in other One Health Scenarios, for example in detecting a bacterial hazard at a particular design prevalence in a food product.

In the design section of this framework, we recommend a robust method of objective setting and highlight this as a reference point for all subsequent activities in the framework. We also emphasize the importance of identifying all the stakeholders acting within the OBS system and demonstrate how stakeholder engagement can guide the design of successful surveillance systems with their expertise and knowledge. We recommend the EpiTools calculator for determining sample size (29) in our worked examples. Later in the design section, we describe a method for estimating the costs of the available test options, helping predict the feasibility of implementing the chosen test within the available surveillance budget.

In the implementation section, we show how systems mapping can be used to visualize the steps and stakeholders involved in surveillance, facilitating clear communication of the intended system design to all relevant stakeholders from an early stage. Later, we highlight the importance of left shift and operational risk analysis to effective project implementation.

The evaluation section described in this framework first establishes whether the stated objectives of the system are still relevant to the contemporary disease and legislative context. Then, the flexibility of the system to adaptation and change is analyzed to provide a holistic view of the relationships between system components and the system’s capacity for change. By applying technical evaluation tools such as EpiTools, we can assess whether the chosen prevalence estimations and sample sizes remain accurate to the true disease situation. This provides an indication of whether individual surveillance streams should be upscaled or downscaled to meet the required output of the system. Along with a technical performance assessment, this guidance provides advice on how to evaluate the human factors within the system through stakeholder evaluation. Financial viewpoints are considered in the cost-effectiveness analysis section. This provides an example evaluation method for multiple testing options. In completing the full evaluation, the technical, human, economic, and practical elements of the system can be visualized in the wider context of the current disease situation.

However, there are limitations to some of the analyses described. For example, because of the variation across laboratories, countries, and sectors, the CEA did not consider the implementation costs of *changing* the testing type used. These are the additional costs required to move from one testing type to another, including the cost of retraining staff, and purchasing new equipment. Including implementation costs would provide a better understanding of the real costs of applying different test types. Any future expansions to this work could integrate the payback times for different tests following initial investment in them over a temporal dimension. This could say, for example, that moving to a PCR and fecal sample-based testing regime, while it would cost £3 M investment, would pay itself back in savings from reduced year-on-year sample collection and material costs in 10 years. Under this framework it was not possible to quantify the implementation costs of new training and equipment without knowing the existing laboratory capacity. Thus, to keep the analysis generic to a range of end-users, this aspect was not included.

Additionally, because this guidance is designed for OBS systems only, the recommendations it provides are more tailored than other surveillance evaluation tools such as SERVAL and RISKSUR EVA, which are generic to all forms of surveillance (4, 5). Its narrower scope provided an opportunity to ground this framework to worked examples that highlight immediate practical recommendations rather than top-level areas for improvement. However, we acknowledge that some elements of the framework may be prescriptive.

For instance, EpiTools is referenced throughout the guidance, without consideration of other epidemiological calculators. The calculator by Iowa state university, for instance, could equally be used for sample size and probability of detection calculation (76). We chose EpiTools for the examples because of its broad range of available analysis applications, including sample size estimations using both hypergeometric and binomial approaches and true prevalence estimations using Bayesian and pooled computational approaches. This range of analyses makes it applicable to OBS systems with large or small population sizes, and with a broad design prevalence range. In addition, the tool is free and has had usage across several published articles, making it readily accessible to analysts from a range of backgrounds (77–79).

Many of the ideas in the implementation section of this framework are tied to systems engineering practices. These have a good track record of use across a range of science and technology-focused projects (19, 80). However, several analyses in this framework could be conducted differently. For example, while causal loop diagrams have been used in a wide range of disciplines to represent dynamic systems (23, 44), analysts could equally use retrospective approaches for flexibility analysis as in the guidelines for evaluating public health surveillance systems produced by the United States Centers for Disease Control (81). We also acknowledge that not all sections of this framework will be relevant to all users and that, depending on the context of its users, there may be gaps that require additional research. This is expected given the broad scope of OBS in different situations, and as such this guidance should be considered alongside other training and literature from other sources. Nevertheless, we believe that the approaches described here encourage a holistic outlook on OBS systems throughout. Above all, they encourage extensive stakeholder engagement, not only with end users, but also with delivery and governance teams. We hope this framework will encourage cross-disciplinary implementations of OBS systems and thereby improve their performance and sustainability.

In summary, this framework provides a range of relevant activities and recommendations for the design, implementation, and evaluation of output-based surveillance systems. It is a holistic toolkit with applications from setting the objectives of a new system to analyzing the cost-performance of an established system. Not all sections will be applicable to all end users. However, its promotion of systems thinking, and stakeholder participation makes it a valuable tool in the cross-disciplinary implementation of OBS.

## Data availability statement

The original contributions presented in the study are included in the article/Supplementary material, further inquiries can be directed to the corresponding author.

## Author contributions

SR wrote the first draft of the manuscript. SR, MK, RD, AS, AZ-B, and VH wrote sections of the manuscript. All authors contributed to the article and approved the submitted version.

## Funding

This work, carried out as part of the MATRIX project (Promoting One Health in Europe through joint actions on foodborne zoonoses, antimicrobial resistance and emerging microbiological hazards) has received funding from the European Union’s Horizon 2020 research and innovation programme under Grant Agreement No 773830 and from the Department for Environment, Food and Rural Affairs (DEFRA) UK. Research at the National Veterinary Research Institute (PIWet), Poland, was also partially supported by the Polish Ministry of Education and Science from the funds for science in the years 2018–2022 allocated for the implementation of co-financed international projects.

## Conflict of interest

The authors declare that the research was conducted in the absence of any commercial or financial relationships that could be construed as a potential conflict of interest.

## Publisher’s note

All claims expressed in this article are solely those of the authors and do not necessarily represent those of their affiliated organizations, or those of the publisher, the editors and the reviewers. Any product that may be evaluated in this article, or claim that may be made by its manufacturer, is not guaranteed or endorsed by the publisher.

## Footnotes

^1^ www.scopus.com

^2^ www.ncbi.nlm.nih.gov/pubmed

## References

[1] Bordier M Uea-Anuwong T Binot A Hendrikx P Goutard F. Characteristics of One Health surveillance systems: A systematic literature review. Prev Vet Med. (2018) 181. doi: 10.1016/j.prevetmed.2018.10.005 30528937

[2] Cameron AR. (2012) The consequences of risk-based surveillance: Developing output-based standards for surveillance to demonstrate freedom from disease. Prev Vet Med. (2012) 105:280–6. doi: 10.1016/j.prevetmed.2012.01.009, PMID: 22305852

[3] Meletis (2022) Output-based methodological approaches for substantiating freedom from infection. Accessed 2022, Publisher/Project: SOUND Control, https://sound-control.eu/wp-content/uploads/2021/12/Deliverable-3.1.-Output-based-methodological-approaches-for-substantiating-freedom-from-infection.pdf 10.3389/fvets.2024.1337661 PMC10977073 38550781

[4] Drewe J Hoinville L Cook A Floyd T Gunn G Stärk K. SERVAL: a new framework for the evaluation of animal health surveillance. Transbound Emerg Dis. (2015) 62:33–45. doi: 10.1111/tbed.12063, PMID: 23414450

[5] Peyre M Hoinville L Njoroge J Cameron A Traon D Goutard F . The RISKSUR EVA tool (Survtool): a tool for the integrated evaluation of animal health surveillance systems. Prev Vet Med. (2019) 173:104777. doi: 10.1016/j.prevetmed.2019.104777, PMID: 31731037

[6] Ausvet. Sample size to achieve specified population level (or herd, flock, cluster, etc) sensitivity. AUSVET. (2022). Available at: https://epitools.ausvet.com.au/freedomss (Accessed May 23, 2022)

[7] Henaux V Tegegne H Bogaardt C Lailler R Collineau L Prada J. Deliverable D-JIP-MATRIX-WP4.2 OH-EpiCap tool and tutorial. Zenodo. (2022)

[8] Costa L Duarte EL Knific T Hodnik JJ van Roon A Fourichon C . Standardizing output-based surveillance to control non-regulated cattle diseases: aspiring for a single general regulatory framework in the European Union. Prev Vet Med. (2020) 183:105130. doi: 10.1016/j.prevetmed.2020.105130, PMID: 32920493 PMC7446655

[9] Horigan V. Inventory of previous work and current practice using output based standards. Deliverable D-WP3.2: Guidelines for the design, implementation, and evaluation of official controls within the food sector using output-based standards (OBS) Zenodo (2022).

[10] Alban L Rugbjerg H Petersen JV Nielsen LR. Comparison of risk-based versus random sampling in the monitoring of antimicrobial residues in Danish finishing pigs. Prev Vet Med. (2016) 128:87–94. doi: 10.1016/j.prevetmed.2016.04.007, PMID: 27237394

[11] Foddai A. Nielsen L. R. Willeberg P. Alban L. (2015). Comparison of output-based approaches used to substantiate bovine tuberculosis free status in Danish cattle herds, 121, 21–29 doi: 10.1016/j.prevetmed.2015.05.005, PMID: 26036341

[12] Reber A Reist M Schwermer H. Cost-effectiveness of bulk-tank milk testing for surveys to demonstrate freedom from infectious bovine rhinotracheitis and bovine enzootic leucosis in Switzerland. Schweizer Archiv Fur Tierheilkunde. (2012) 154:189–97. doi: 10.1024/0036-7281/a000329, PMID: 22547334

[13] Rutten N Gonzales JL Elbers AR Velthuis AG. Cost analysis of various low pathogenic avian influenza surveillance systems in the Dutch egg layer sector. PLoS One. (2012) 7:e33930. doi: 10.1371/journal.pone.0033930, PMID: 22523543 PMC3327686

[14] Foddai A Floyd T Mcgiven J Grace K Evans S. Evaluation of the English bovine brucellosis surveillance system considering probability of disease introduction and non-random sampling. Prev Vet Med. (2020) 176:104927. doi: 10.1016/j.prevetmed.2020.104927, PMID: 32135412

[15] Mastin AJ Gottwald TR Van Den Bosch F Cunniffe NJ Parnell S. Optimising risk-based surveillance for early detection of invasive plant pathogens. PLoS Biol. (2020) 18:e3000863. doi: 10.1371/journal.pbio.3000863, PMID: 33044954 PMC7581011

[16] de Vos CJ Saatkamp H Huirne R. Cost-effectiveness of measures to prevent classical swine fever introduction into the Netherlands. Prev Vet Med. (2005) 70:235–56. doi: 10.1016/j.prevetmed.2005.04.001, PMID: 15927286

[17] Hadorn DC Racloz V Schwermer H Stärk KD. Establishing a cost-effective national surveillance system for bluetongue using scenario tree modelling. Vet Res. (2009) 40:1–14. 19607784 10.1051/vetres/2009040 PMC2736541

[18] Dewar R. (2021). System analysis of detection of outbreaks. COHESIVE: One Health Structure In Europe, Deliverable-D-JIP2-D3.5.

[19] Emes MR Smith A James AM Whyndham MW Leal R Jackson SC. 8.1.2 principles of systems engineering management: reflections from 45 years of spacecraft technology research and development at the Mullard space science laboratory. INCOSE Int Symp. (2012) 22:1069–84.

[20] Mendelow A. L. (1981). Environmental scanning--the impact of the stakeholder concept. ICIS 1981 Proceedings. 20, https://aisel.aisnet.org/icis1981/20

[21] COHESIVE consortium Cohesive One health: setting up a risk analysis system for zoonoses. (2022). Available at: https://www.ohras.eu/page/home (Accessed May 17, 2022).

[22] Dewar R. (2021). Review of economic analyses conducted on foodborne Zoonoses. DeliverableD-JIP2-3.8.0.

[23] Tip T. Guidelines for drawing causal loop diagrams. Systems Thinker. (2011) 22:5–7.

[24] Stevenson M. Sergeant E. (2022). Design and analysis of disease surveillance programs using epiR. Available at: https://cran.r-project.org/web/packages/epiR/vignettes/epiR_surveillance.html (Accessed October 24, 2022).

[25] Paoli BHL Shah G. Confidence intervals in public health Office of Public Health Assessment, Utah Department of Health (2002).

[26] Rahmatian S. The hierarchy of objectives: toward an integrating construct in systems science. Syst Res. (1985) 2:237–45. doi: 10.1002/sres.3850020307

[27] EFSA (European Food Safety Authority) Beltran-Beck B Zancanaro G. Scientific report on the assessment of Echinococcus multilocularis surveillance reports submitted in2017 in the context of Commission Regulation (EU) No 1152/2011. EFSA Journal 2017. (2017) 15:5051–75. doi: 10.2903/j.efsa.2017.5051 PMC7010048 32625344

[28] Friedman A Miles S. Stakeholders: Theory and Practice. UK: Oxford University Press (2006).

[29] Sergeant E. Epitools epidemiological calculators. (2018). Canberra, Australia. Available at: http://epitools.ausvet.com.au.

[30] Mathews F Kubasiewicz L Gurnell J Harrower C Mcdonald RA Shore R. A review of the population and conservation status of British mammals: Technical summary. Natural England (2018).

[31] European Food Safety Authority. Annual assessment of *Echinococcus multilocularis* surveillance reports submitted in 2020 in the context of commission delegated regulation (EU) 2018/772. EFSA J. (2021) 19:e06382. doi: 10.2903/j.efsa.2021.6382 33537068 PMC7845509

[32] European Food Safety Authority. Annual assessment of *Echinococcus multilocularis* surveillance reports submitted in 2021 in the context of commission delegated regulation (EU) 2018/772. EFSA J. (2021) 19:e06945. doi: 10.2903/j.efsa.2021.6945 34824646 PMC8600939

[33] European Food Safety Authority. A framework to substantiate absence of disease: the risk based estimate of system sensitivity tool (RiBESS) using data collated according to the EFSA standard sample description – an example on *Echinococcus multilocularis*. EFSA. (2012) 9:366E.

[34] Emes M. Smith A. James A. (2014). ‘Left-shift’ vs the time value of money. European Food Safety Authority, Italy.

[35] Risksur Consortium. Risk-based animal health surveillance systems framework. (2015). Available at: https://www.fp7-risksur.eu/ (Accessed March 08, 2023).

[36] Weilkiens T. Chapter 1: The V model. Systems engineering with SysML/UML: Modeling, analysis, design Elsevier (2011). USA: Morgan Kaufmann.

[37] Davison N. (2016). The change control process. In: Resources, U. P. M. (ed.).

[38] Devi TR Reddy VS. Work breakdown structure of the project. Int J Eng Res Appl. (2012) 2:683–6.

[39] Lester A. Project management, planning and control: Managing engineering, construction and manufacturing projects to PMI, APM and BSI standards Elsevier (2006).

[40] Wilson JM. Gantt charts: a centenary appreciation. Eur J Oper Res. (2003) 149:430–7. doi: 10.1016/S0377-2217(02)00769-5

[41] Lavanya N Malarvizhi T. Risk analysis and management: a vital key to effective project management. PMI® global congress. Sydney: Project Management Institute (2008).

[42] Institution Of Civil Engineers. Risk analysis and management for projects ICE Publishing (2002).

[43] Kobusingye B Mungatu J Mulyungi P. Influence of stakeholders involvement on project outcomes. A case of water, sanitation, and hygiene (wash) project in Rwanda. Eur J Bus Soc Sci. (2017) 6:195–206.

[44] Haraldsson HV. Introduction to system thinking and causal loop diagrams. Lund: Department of Chemical Engineering, Lund University (2004).

[45] APHA carcass collection coordinator Interview with APHA carcass collection coordinator. (2022).

[46] Macdiarmid S. Future options for brucellosis surveillance in New Zealand beef herds. N Z Vet J. (1988) 36:39–42. doi: 10.1080/00480169.1988.35472, PMID: 16031432

[47] The Forest Data Bank. (2022). Available at: https://www.bdl.lasy.gov.pl/portal/en

[48] Kirjušina M Deksne G Marucci G Bakasejevs E Jahundoviča I Daukšte A . A 38-year study on *Trichinella* spp. in wild boar (*Sus scrofa*) of Latvia shows a stable incidence with an increased parasite biomass in the last decade. Parasit Vectors. (2015) 8:137–8. doi: 10.1186/s13071-015-0753-1 25886306 PMC4351677

[49] Danish Centre for Environment and Energy. (2022). Available at: https://dce.au.dk/en/

[50] European Food Safety Authority. Assessment of *Echinococcus multilocularis* surveillance reports submitted in 2015 in the context of commission regulation (EU) no 1152/2011. EFSA J. (2015) 13:4310. doi: 10.2903/j.efsa.2015.4310 PMC13189517 42169848

[51] Şuteu O Mihalca AD Paştiu AI Györke A Matei IA Ionică A . Red foxes (*Vulpes vulpes*) in Romania are carriers of toxoplasma gondii but not *Neospora caninum*. J Wildl Dis. (2014) 50:713–6. doi: 10.7589/2013-07-167, PMID: 24807364

[52] Romanian National Institute of Statistics. (2008). Available at: http://www.insse.ro/cms/files/statistici/comunicate/com_anuale/fond%20cinegetic/Fondcinegetic_2008.pdf

[53] Kauhala K. (2007). Paljonko Suomessa on pienpetoja?

[54] Hayden TJ Harrington R. Exploring Irish mammals Town House (2000).

[55] Marnell F Kingston N Looney D. Ireland red list no. 3: Terrestrial mammals. Dublin: National Parks and Wildlife Service, Department of the Environment, Heritage and Local Government (2009).

[56] Defra Department For Environment, F. R. A. & Agency, A. A. A. P. H. (2019). *Echinococcus multilocularis*: How to spot and report the disease. Available at: https://www.gov.uk/guidance/echinococcus-multilocularis-how-to-spot-and-report-the-disease (Accessed September 30, 2022).

[57] Sviland S Johansen T Klevar S Valheim M Jonsson M. Surveillance programmes for terrestrial and aquatic animals in Norway. Annu Rep. (2014)

[58] Karamon J Kochanowski M Sroka J Cencek T Różycki M Chmurzyńska E . The prevalence of *Echinococcus multilocularis* in red foxes in Poland—current results (2009–2013). Parasitol Res. (2014) 113:317–22. doi: 10.1007/s00436-013-3657-z, PMID: 24221887 PMC3898514

[59] Bagrade G Šnábel V Romig T Ozoliņš J Hüttner M Miterpáková M . *Echinococcus multilocularis* is a frequent parasite of red foxes (*Vulpes vulpes*) in Latvia. Helminthologia. (2008) 45:157–61. doi: 10.2478/s11687-008-0032-1

[60] Combes B Comte S Raton V Raoul F Boué F Umhang G . Westward spread of *Echinococcus multilocularis* in foxes, France, 2005-2010. Emerg Infect Dis. (2012) 18:2059–62. doi: 10.3201/eid1812.120219, PMID: 23171707 PMC3557902

[61] Berke O Romig T Von Keyserlingk M. Emergence of *Echinococcus multilocularis* among red foxes in northern Germany, 1991–2005. Vet Parasitol. (2008) 155:319–22. doi: 10.1016/j.vetpar.2008.05.017, PMID: 18583056

[62] Enemark HL Al-Sabi MN Knapp J Staahl M Chríel M. Detection of a high-endemic focus of *Echinococcus multilocularis* in red foxes in southern Denmark, January 2013. Euro Surveill. (2013) 18:20420. 23515060 10.2807/ese.18.10.20420-en

[63] Sréter T Széll Z Sréter-Lancz Z Varga I. *Echinococcus multilocularis* in northern Hungary. Emerg Infect Dis. (2004) 10:1344–6. doi: 10.3201/eid1007.031027, PMID: 15338552 PMC3323332

[64] Sikó SB Deplazes P Ceica C Tivadar CS Bogolin I Popescu S . *Echinococcus multilocularis* in South-Eastern Europe (Romania). Parasitol Res. (2011) 108:1093–7. doi: 10.1007/s00436-010-2150-1, PMID: 21085988

[65] Hanosset R Saegerman C Adant S Massart L Losson B. *Echinococcus multilocularis* in Belgium: prevalence in red foxes (*Vulpes vulpes*) and in different species of potential intermediate hosts. Vet Parasitol. (2008) 151:212–7. doi: 10.1016/j.vetpar.2007.09.024, PMID: 18164551

[66] Moks E Saarma U Valdmann H. *Echinococcus multilocularis* in Estonia. Emerg Infect Dis. (2005) 11:1973–4. doi: 10.3201/eid1112.050339, PMID: 16485495 PMC3367629

[67] Otero-Abad B Armua-Fernandez MT Deplazes P Torgerson PR Hartnack S. Latent class models for *Echinococcus multilocularis* diagnosis in foxes in Switzerland in the absence of a gold standard. Parasit Vectors. (2017) 10:1–14. 29258612 10.1186/s13071-017-2562-1 PMC5737983

[68] Hofer S Gloor S Müller U Mathis A Hegglin D Deplazes P. High prevalence of *Echinococcus multilocularis* in urban red foxes (*Vulpes vulpes*) and voles (*Arvicola terrestris*) in the city of Zürich. Switzerland Parasitol. (2000) 120:135–42. doi: 10.1017/S0031182099005351 10726275

[69] EFSA (European Food Safety Authority) Zancanaro G. Annual assessment of *Echinococcus multilocularis* surveillance reports submitted in 2021 in the context of Commission Delegated Regulation (EU) 2018/772. EFSA Journal 2021. (2021) 19:57. doi: 10.2903/j.efsa.2021.6945 PMC8600939 34824646

[70] European Union Reference Laboratory for Parasites (2022) *Proficiency testing*, Istituto Superiore di Sanità. https://www.iss.it/en/web/iss-en/eurlp-proficiency-testing (Accessed October, 2022).

[71] Eckert J. Predictive values and quality control of techniques for the diagnosis of Echinococcus multilocularis in definitive hosts. Acta tropica. (2003) 85:157–63. doi: 10.1016/S0001-706X(02)00216-4 12606092

[72] Knapp J Millon L Mouzon L Umhang G Raoul F Zeinaba S . Real time PCR to detect the environmental faecal contamination by Echinococcus multilocularis from red fox stools. (2014). 10.1016/j.vetpar.2013.12.023 24484767

[73] Maksimov P Isaksson M Schares G Romig T Conraths F. Validation of different PCR-based protocols for the detection of Echinococcus multilocularis DNA in the final host using the Intestinal Scraping Technique as a reference. Food and Waterborne Parasitology. (2019) 15:e00044. doi: 10.1016/j.fawpar.2019.e00044 32095616 PMC7034050

[74] Central Unit for Sequencing and PCR (CUSP) RE:qPCR reagents costs. (2022).

[75] APHA parasitology lead Interview with APHA parasitology lead. (2022).

[76] Iowa State University. Sample size calculators. (2022). Available at: https://fieldepi.research.cvm.iastate.edu/calc/ (Accessed October 26, 2022).

[77] Charan J Kantharia N. How to calculate sample size in animal studies? J Pharmacol Pharmacother. (2013) 4:303–6. doi: 10.4103/0976-500X.119726, PMID: 24250214 PMC3826013

[78] Laurin E Bradshaw J Hawley L Gardner IA Garver K Johnson SC . Importance of sample size for estimating prevalence: a case example of infectious hematopoietic necrosis viral RNA detection in mixed-stock Fraser River sockeye salmon (Oncorhynchus nerka), British Columbia, Canada. Can J Fish Aquat Sci. (2021) 78:589–98. doi: 10.1139/cjfas-2020-0279

[79] Villarta, Jr RL Asaad AS. Sample size determination in an epidemiologic study using the EpiTools web-based calculator. Acta Med Philipp. (2014) 48. doi: 10.47895/amp.v48i1.1192

[80] Leal R. (2020). The foundations of systems engineering. In: Engineering, U. C. F. S. (Ed.). London.

[81] German RR Horan JM Lee LM Milstein B Pertowski CA. Updated guidelines for evaluating public health surveillance systems; recommendations from the guidelines working group US CDC (2001). 18634202

